# Reducing errors in health care: cost-effectiveness of multidisciplinary team training in obstetric emergencies (TOSTI study); a randomised controlled trial

**DOI:** 10.1186/1471-2393-10-59

**Published:** 2010-10-08

**Authors:** Joost van de Ven, Saskia Houterman, Rob AJQ Steinweg, Albert JJA Scherpbier, Willy Wijers, Ben William J Mol, S Guid Oei

**Affiliations:** 1 Department of Obstetrics and Gynaecology, Máxima Medical Centre Eindhoven-Veldhoven, the Netherlands; 2 MMC Academy, Máxima Medical Centre Eindhoven-Veldhoven, the Netherlands; 3 Managing director MedSim (Medical Education and Simulation Centre), Máxima Medical Centre Eindhoven-Veldhoven, the Netherlands; 4 Educational Institute, Faculty of Health, Medicine and Life Sciences, Maastricht University, the Netherlands; 5 Department of Obstetrics and Gynaecology, Academic Medical Centre Amsterdam, the Netherlands; 6 Department of Electrotechnical Engineering, Eindhoven University of Technology, Eindhoven, the Netherlands

## Abstract

**Background:**

There are many avoidable deaths in hospitals because the care team is not well attuned. Training in emergency situations is generally followed on an individual basis. In practice, however, hospital patients are treated by a team composed of various disciplines. To prevent communication errors, it is important to focus the training on the team as a whole, rather than on the individual. Team training appears to be important in contributing toward preventing these errors. Obstetrics lends itself to multidisciplinary team training. It is a field in which nurses, midwives, obstetricians and paediatricians work together and where decisions must be made and actions must be carried out under extreme time pressure.

It is attractive to belief that multidisciplinary team training will reduce the number of errors in obstetrics. The other side of the medal is that many hospitals are buying expensive patient simulators without proper evaluation of the training method. In the Netherlands many hospitals have 1,000 or less annual deliveries. In our small country it might therefore be more cost-effective to train obstetric teams in medical simulation centres with well trained personnel, high fidelity patient simulators, and well defined training programmes.

**Methods/design:**

The aim of the present study is to evaluate the cost-effectiveness of multidisciplinary team training in a medical simulation centre in the Netherlands to reduce the number of medical errors in obstetric emergency situations. We plan a multicentre randomised study with the centre as unit of analysis. Obstetric departments will be randomly assigned to receive multidisciplinary team training in a medical simulation centre or to a control arm without any team training.

The composite measure of poor perinatal and maternal outcome in the non training group was thought to be 15%, on the basis of data obtained from the National Dutch Perinatal Registry and the guidelines of the Dutch Society of Obstetrics and Gynaecology (NVOG). We anticipated that multidisciplinary team training would reduce this risk to 5%. A sample size of 24 centres with a cluster size of each at least 200 deliveries, each 12 centres per group, was needed for 80% power and a 5% type 1 error probability (two-sided). We assumed an Intraclass Correlation Coefficient (ICC) value of maximum 0.08.

The analysis will be performed according to the intention-to-treat principle and stratified for teaching or non-teaching hospitals.

Primary outcome is the number of obstetric complications throughout the first year period after the intervention. If multidisciplinary team training appears to be effective a cost-effective analysis will be performed.

**Discussion:**

If multidisciplinary team training appears to be cost-effective, this training should be implemented in extra training for gynaecologists.

**Trial Registration:**

The protocol is registered in the clinical trial register number NTR1859

## Background

In the United States 44,000 to 98,000 patients die in hospitals each year as a result of medical errors that possibly could have been prevented [1]. Of the fatal errors 75% takes place in the category 'failure to rescue'. Many of these avoidable medical errors arise by communication errors or because the care team has not anticipated on each other. Training in how to act in emergency situations happens generally on individual basis. In practice, however, a hospital patient is treated by a team of more disciplines and therefore the training must be aimed at the whole team. Obstetrics lends itself to multidisciplinary team training. It is a field in which various professionals work together and where decisions must be made and actions must be carried out under extreme time pressure. An ongoing Dutch enquiry into term perinatal deaths shows that in 30% of cases substandard care factors are present (Bruinse 2008, personal communication). The rate of claims increased, but the most pronounced rise has been in the rate of claims arising from obstetrics and gynaecology. Every year approximately 50% of the National Health Service (United Kingdom) litigation bill of 400 million pound relates to claims arising from obstetrics and gynaecology [2]. In a large British study it has been shown that regular team training in obstetric emergencies results in a 50% reduction of poor perinatal outcome [3].

Crew resource management (CRM) is derived from the aviation industry. Both the Institute of Medicine and the Healthcare Research and Quality suggest that patient safety can be improved by introducing CRM in health care. It has been shown that giving team training to clinical teams leads to improvements in dealing with fatigue, teambuilding, communication, recognising dangerous situations, decision-making and providing feedback [4,5].

The development of patient simulators began in anaesthesiology at the end of the 1980s [6-8]. The first simulators consisted of relatively simple interactive software programs that were played on a computer screen. Later, these software programs were integrated in real-life dummies that were operated by an instructor. The most advanced dummies are model-driven. These high fidelity patient simulators can be fully programmed to simulate a certain acute disorder. The scenarios can be applied to the specific target group. Participants can be tested on their individual clinical skills and competency to work together under pressure as a team. What are the advantages of team training of health care professionals in a medical simulation centre?

- Training of health care teams in emergency situations promotes cooperation and reduces the number of communication errors [9,10];

- Training in a medical simulation centre offers the opportunity to train rare emergency scenarios under standardised conditions and give targeted feedback on functioning as individual and team;

- Acceptance of team training results in a culture that devotes more attention to patient safety [11].

Against these advantages there are also some disadvantages:

- The costs of introducing simulation training strongly depend on the objective of the simulation, the intended target group and the applied technology. Practicing on personal computers and low-fidelity phantoms is relatively inexpensive. Investment in high-fidelity patient simulators is substantial, but cheaper than practicing on laboratory animals [12];

- New training curricula with expensive patient simulators have been developed for obstetric teams and some have already been introduced in Dutch hospitals without proper evaluation of effectiveness and costs of the training. A survey among all Dutch departments of obstetrics in January 2008 revealed that 90% of the responders had the intention to start team training with patient simulators without any clear description of the content of the training;

- Local training of obstetrical teams might be even effective as training in a simulation centre [13]. However, that study was performed in six hospitals in the United Kingdom with delivery rates ranging from 2,500 to 4,600 per annum. Dutch obstetrical departments are relatively small. In the Netherlands delivery rates range from 500 to 2,500 per year. Many hospitals have less than 1,000 deliveries per annum. Therefore it will be complicated to find enough well educated instructors for local hospital training and reach a high level of training which might lead to less effectiveness.

### Relevance

The value of team training in a simulated environment has been recognised in the USA. There are hundreds of medical simulation centres with high fidelity patient simulators. The Riverside Methodist Hospital in Ohio, a highly advanced medical simulation centre, was recently opened. This centre includes three departments:

- A virtual care unit, consisting of an operating room, a trauma room, an intensive care, a surgery room and an emergency room;

- A skills laboratory, consisting of laboratories to learn laparoscopic skills, suturing, catheterisation, clinical skills and patient examination rooms;

- Multimedia conference areas for interactive learning.

Medical simulation centres have also been put in use in the UK, Germany and Scandinavia. The Society for Simulation in Healthcare (SSH) in the United States and the Society in Europe for Simulation Applied to Medicine (SESAM) are growing exponentially and work well together. The Máxima Medical Centre (MMC) in Eindhoven-Veldhoven has taken the initiative in the Netherlands in applying team training using practical medical simulation. Medical simulation is an important component of the 10 years research plan of the collaboration on scientific research between Máxima Medical Centre and the University of Technology Eindhoven (TU/e). In the Medical Education and Simulation Centre (medsim) in Eindhoven all multidisciplinary teams that are engaged in emergency care are trained in a simulation setting that resembles reality as closely as possible. Commercially available high fidelity patient simulators are used. In cooperation with the University of Technology Eindhoven and the University of Porto, work is also being performed on improving simulators and developing new high fidelity patient simulators in the field of neonatology and obstetrics.

For the technological improvement of birthing simulations a subsidy has been recently obtained from the Stimulus foundation (European foundation for regional development) by MMC in association with the University of Technology Eindhoven and the European Design Centre. The development of programs for the medical education and simulation centre (medsim) is made possible by support of Máxima Medical Centre and Dutch health insurance companies VGZ and CZ.

Initially, a start was made with a study in the delivery rooms by Oei et al. in 2006. Six emergency scenarios were used for the study. The teams were comprised of a gynaecologist or a gynaecology resident, a midwife and a nurse or assistant midwife. All instructors and facilitators received special training in crew resource management. Special childbirth simulators are used. The Noelle™ (Gaumard, Miami Florida) maternal childbirth simulator is an interactive manikin that can simulate diverse obstetric emergency scenarios guided by instructors. Baby Hal™ (Gaumard, Miami Florida) is an interactive wireless newborn simulator, and Prompt Birthing Trainer™ (Limbs & Things, Bristol UK) is an interactive birthing simulator for shoulder dystocia with force feedback. Aside from knowledge and skills, cooperation and communication between the various team members is monitored for the assessment. Standardised questionnaires were used for the evaluations. The results showed that all participants were positive about the team training. Individual knowledge, skills, cooperation and communication between the team members improved. The results confirmed the positive experience of other researchers [9,13].

In a large British retrospective cohort study it has been shown that team training in obstetric emergencies results in a reduction of poor perinatal outcome [3]. In a large study where 19,460 neonates were involved, the introduction of obstetric emergencies training courses resulted in a reduction of 49% (from 86.6 to 44.6 per 10,000 births) of neonates born with low Apgar scores (5-minutes <6) and reduction of 50% of infants with hypoxic ischemic encephalopathy (from 27.3 to 13.6 per 10,000 births) [3].

## Methods/Design

The proposed research concerns a multicentre randomised controlled trial of obstetric departments in the Netherlands. These obstetric departments will be randomly assigned to receive multidisciplinary team training in a medical simulation centre or to a control arm without any team training.

### Recruitment

We invited several teaching and non-teaching hospitals in the Netherlands to participate in this randomised study. We excluded hospitals which already have frequently multidisciplinary team training for its care workers, as defined by more than once every year. Randomisation will be performed through a database which is hosted at the Academic Medical Centre (AMC) in Amsterdam. Randomisation will be 1:1 for intervention and control group and it will be stratified for teaching or non-teaching hospital because we expect differences in outcomes in teaching and non-teaching hospitals.

### Hypothesis

Multiprofessional, obstetric emergency training in a medical simulation centre with high fidelity patient simulators and well educated instructors leads to a reduction of perinatal and maternal morbidity.

Questions to be answered:

1. Does medical simulation of obstetric teams in a medical simulation centre reduce the number of errors in acute situations?

2. What is the influence of medical simulation trainings of obstetric teams in a medical simulation centre on the perinatal outcome?

3. What are the costs of medical simulation trainings of obstetric teams in a medical simulation centre?

4. What is the cost-effectiveness of medical simulation trainings of obstetric teams in a medical simulation centre?

### Intervention

The intervention group will have multidisciplinary team training in a medical simulation centre. These team trainings are given by specially trained instructors and facilitators (gynaecologists, communication experts and psychologists). All instructors and facilitators are trained in crew resource management. To make the context of the training lifelike these take place in an entirely reconstructed delivery room. State of the art high fidelity patient simulators will be used. Fetal distress (including a cardiotocogram training program), postpartum haemorrhage, eclampsia, umbilical cord prolapse and shoulder dystocia scenario's are practiced on the birthing simulator Noelle™ (Gaumard Miami, Florida) and Prompt Birthing Trainer™ (Limbs & Things, Bristol), perimortem caesarean section on the emergency care simulator ECS™ (Meti, Sarasota, Florida), and resuscitation of asphyxiated infants on the neonatal simulator (Baby Hal™, Gaumard, Miami, Florida). For hospitals randomised to receive the team training, the interventions will be planned. Depending on the size of the department it concerns 3 up to 10 sessions. A team will consist of a group of about 6 care workers in means of one gynaecologist and one or more residents, midwifes and nurses. For one hospital all sessions are carried out in a timeframe with a maximum of 4 weeks. This means that several teams of the same hospital will be trained. In the real life setting in their own hospitals there will be a different composition of team members but every delivery room care worker has been trained in the simulation centre.

The team training will take place at the medical education and simulation centre in Eindhoven (medsim) where the following scenarios will be trained: Fetal distress including CTG analysis, shoulder dystocia, severe postpartum haemorrhage, eclampsia, umbilical cord prolapse and perimortem caesarean section. The scenarios are based on national and international accepted guidelines by NVOG (The Dutch Society for Obstetrics and Gynaecology) and MOET (Managing Obstetric Emergencies & Trauma). Each scenario starts with a briefing session in a simulated emergency room of 10 minutes in which the case is presented by means of a video play on a wide screen. The team will then move to the simulated delivery room. In the delivery room they have to treat the patient. The patient is a an advanced interactive birthing simulator. The facilitator gives wireless instructions to the robot from the control room. The whole session is videotaped. The simulation session will take 20 minutes. Then the team will move to the briefing room. The facilitators will give feedback on teamwork and skills using video recording. The debriefing session will take about 30 minutes.

There will be an effect measurement after six months following the team training, by in-situ training in all participating hospitals, including the control arm.

### Outcome measures

Primary outcome is the number of obstetric complications throughout the first year after the intervention. Obstetric complications will be defined as the number of neonates with perinatal asphyxia (Apgar score 5-minutes <7 and/or arterial umbilical pH < 7.05, hypoxic ischemic encephalopathy (HIE), number of newborns with damage caused by shoulder dystocia (e.g. lesion of brachial plexus, clavicle fracture), number of women with eclampsia, number of women with severe post partum haemorrhage (blood transfusion >4 packed cells, embolisation, hysterectomy). Shoulder dystocia is hereby described as every additional manoeuvre for successful alleviation.

Sub analysis will be performed to identify possible changes in subgroups. These groups concern term infants (gestation over 37 weeks), singleton pregnancies and cephalic presentation at birth.

Secondary outcomes are perinatal and maternal mortality, divided by causes of death.

These complications will be obtained from the regular obstetric recordings after informed consent from the participating hospitals (with exception of damage due to shoulder dystocia and severe postpartum haemorrhage, these data will be registered separately). Before the start of the project indicators will be developed to evaluate patient safety, teamwork and human factors. These indicators will be registered in a subgroup of the participating hospitals. This group will consist of 100 obstetric complications equally distributed in the intervention and control group.

### Economic evaluation

The trial results will be incorporated in a cost-effectiveness analysis to compare the costs and effects of multidisciplinary team training in a medical simulation centre (experimental strategy) versus no such training (reference strategy).

#### Cost analysis

The process of care is distinguished into two cost stages: delivery/childbirth and postnatal stage and three cost categories: direct medical costs (all costs in the health care sector), direct non-medical costs (costs outside the health care sector that are affected by health status or health care) and indirect costs of the pregnant woman and her partner (costs of sick leave). For each stage and each cost category, costs are measured as the volumes of resources used multiplied with appropriate valuations (cost-per-unit estimates, fees, national reference prices). Costs during childbirth are dominated by the course of childbirth and type of delivery. Cost volumes in the postnatal stage consist of maternal care (hospitalisation etc.), neonatal care (admission to NICU/neonatology ward, outpatient visits) and primary care. If neonatal health at discharge is suboptimal, further direct medical, direct non-medical and indirect costs may occur. Hence, for these infants, resource use of infants and/or parents is measured during 12 months after childbirth. Volumes of health care resource use are measured prospectively alongside the clinical study in all participating centres as part of the case report form (CRF). Health resource use outside the hospital is recorded by questionnaires. Valuations of direct medical resources are estimated as cost per unit estimates comprising (true economic) costs, i.e. including shares of fixed costs and hospital overheads. Cost per units are estimated for at least one teaching and one non-teaching hospital. An analysis based on reimbursement fees is added. Direct medical volumes outside the hospital and direct non-medical volumes are valued using national reference prices. Indirect costs are quantified but remain unvalued. Study-specific costs are excluded from analysis.

The costs of the implementation intervention will be calculated separately. We will calculate the costs of patient counselling, the costs of education of health care professional as well as the costs of distribution of patient leaflets. The sensitivity of costs and health outcomes for various parameters is tested in sensitivity analysis and visualised in Incremental Cost-Effectiveness Ratio (ICER) graphs and acceptability curves. When the study would show that multidisciplinary team training would be effective, the primary economic analysis will be a cost-effectiveness analysis. If multidisciplinary team training would appear to reduce complications, we will calculate the costs per prevented complication. No power calculations will be performed for the economic evaluation study as it is embedded in a randomised controlled trial.

### Statistics

#### Sample size

The composite measure of poor perinatal and maternal outcome in the non training group was thought to be 15%, on the basis of data obtained from the National Dutch Perinatal Registry and the guidelines of the Dutch Society of Obstetrics and Gynaecology (NVOG). We anticipated that multidisciplinary team training would reduce this risk to 5%. A sample size of 24 centres with a cluster size of each at least 200 deliveries, each 12 centres per group, was needed for 80% power and a 5% type 1 error probability (two-sided). We assumed an Intraclass Correlation Coefficient (ICC) value with a maximum of 0.08. Because there is an uncertainty about the complication rate and perhaps this is lower than expected a sample size of 1000 deliveries per hospital was taken. In conclusion we will try to randomise 24 obstetric departments in the Netherlands with an one year follow-up.

#### Data analysis

Our primary (base-case analyses) will be performed according to the intention-to-treat principle and stratified for teaching or non-teaching hospital. A baseline analysis will be performed to examine the comparability of groups at baseline for both costs and outcomes. To investigate whether data are normally distributed a Kolmogorov-Smirnov test will be performed. Despite the usual skewness in the distribution of costs, the arithmetic means will be generally considered the most appropriate measures to describe cost data. Therefore arithmetic means (and standard deviations) will be presented. In case of skewness of the cost data, non-parametric bootstrapping will be used to test for statistical differences in costs between the intervention and control group. Non-parametric bootstrapping is a method based on random sampling with replacement based on individual data of the participants. The bootstrap replications will be used to calculate 95% confidence intervals around the costs (95% CI), based on the 2.5th and 97.5th percentiles. If cost data are distributed normally, t-tests will be used. The Incremental Cost-Effectiveness Ratio (ICER) will be determined on the basis of incremental costs and effects of team training compared with non-team training. The cost-effectiveness ratio will be stated in terms of costs per outcome rate, the cost-utility ratio will focus on the net cost per QALY (Quality Adjusted Life Year) gained. The robustness of the ICER will be checked by non-parametric bootstrapping (1000 times). Bootstrap simulations will also be conducted in order to quantify the uncertainty around the ICER, yielding information about the joint distribution of cost and effect differences. The bootstrapped cost-effectiveness ratios will be subsequently plotted in a cost-effectiveness plane, in which the vertical line reflects the difference in costs and the horizontal line reflects the difference in effectiveness. The choice of treatment depends on the maximum amount of money that society is prepared to pay for a gain in effectiveness, which is called the ceiling ratio. Therefore, the bootstrapped ICERs will also be depicted in a cost-effectiveness acceptability curve showing the probability that team training is cost-effective using a range of ceiling ratios.

Sensitivity analysis will be performed for the most important variables, i.e. costs, number of complications etcetera. In a scenario analysis we will evaluate what the effect is of variation of the frequency of team training, in a range of once every two years versus once in three months.

## Discussion

There are many avoidable deaths in hospitals because the care team is not well attuned. Training in emergency situations is generally followed on an individual basis. In practice, however, hospital patients are treated by a team composed of various disciplines. To prevent communication errors, it is important to focus the training on the team as a whole, rather than on the individual. Team training appears to be important in contributing toward preventing these errors. Obstetrics lends itself to multidisciplinary team training. It is a field in which nurses, midwives, obstetricians and paediatricians work together and where decisions must be made and actions must be carried out under extreme time pressure.

If multidisciplinary team training appears to be effective, this training should be implemented in extra training for gynaecologists.

## Competing interests

The authors declare that they have no competing interests.

## Authors' contributions

SGO, SH and BWM were involved in conception and design of the study. JvdV, SGO and BWM drafted the manuscript. All authors mentioned in the manuscript are member of the TOSTI-trial group. They participated in the design of the study during several meetings and are local investigators at the participating centres. All authors edited the manuscript and read and approved the final manuscript.

## Pre-publication history

The pre-publication history for this paper can be accessed here:

http://www.biomedcentral.com/1471-2393/10/59/prepub

## References

[B1] CESDI 7th annual report London: Maternal and Child Health Research Consortium, 2000 Committee on Quality Health Care in America. Institute of Medicine. Crossing the quality chasm: a new health system for the 21st century 2001 Washington, DC: National Academy Press

[B2] Department of Health An Organisation with a Memory Report of an expert Group on Learning from adverse Events in the NHS 2000 London: The Stationary Office

[B3] Draycott T Sibanda T Owen L Akande V Winter C Reading S Whitelaw A Does training in obstetric emergencies improve neonatal outcome? BJOG 2006 113 177 182 10.1111/j.1471-0528.2006.00800.x 16411995

[B4] Grogan EL Stiles RA France DJ Speroff T Morris JA Nixon B Gaffney FA Seddon R Pinson CW The impact of aviation-based teamwork training on the attitudes of health-care professionals J Am Coll Surg 2004 199 843 848 10.1016/j.jamcollsurg.2004.08.021 15555963

[B5] Oei SG Koops W van Uytrecht C Porath M Mulders LGM Op elkaar inspelen. Multidisciplinaire teamtraining verbetert patiëntveiligheid Medisch Contact 2006 61 904 906

[B6] Schwid HA A flight simulator for general anesthesia training Comput Biomed Res 1987 20 64 75 10.1016/0010-4809(87)90019-X 3549134

[B7] Chopra V Gesink BJ de Jong J Bovill JG Spierdijk J Brand R Does training on an anaesthesia simulator lead to improvement in performance? Br J Anaesth 1994 73 293 297 10.1093/bja/73.3.293 7946851

[B8] Meurs van WL Good ML Lampotang S Functional anatomy of full-scale patient simulators J Clin Monit 1997 13 317 324 10.1023/A:1007456108111 9338846

[B9] Sachs BP Effects of Teamwork Training on Adverse Outcomes and Process of Care in Labor and Delivery Obstet Gynecol 2007 109 48 55 17197587 10.1097/01.AOG.0000250900.53126.c2

[B10] Leape LL Berwick DM Five years after To Err Is Human: what have we learned? JAMA 2005 293 2384 2390 10.1001/jama.293.19.2384 15900009

[B11] Gaba DM Howard SK Flanagan B Smith BE Fish KJ Botney R Assessment of clinical performance during simulated crises using both technical and behavioral ratings Anesthesiology 1998 89 8 18 10.1097/00000542-199807000-00005 9667288

[B12] Gaba DM The future vision of simulation in health care Qual Saf Health Care 2004 13 Suppl 1 i2 10 10.1136/qshc.2004.009878 15465951 PMC1765792

[B13] Crofts JF Ellis D Draycott TJ Winter C Hunt LP Akande VA Change in knowledge of midwives and obstetric emergency training: a randomised controlled trial of local hospital, simulation centre and teamwork training BJOC 2007 114 1534 1541 10.1111/j.1471-0528.2007.01493.x 17903231

